# Tissue-Specific Gene Expression of Digestive Tract Glands in *Paroctopus digueti*: Insights for Cephalopod Biology and Aquaculture

**DOI:** 10.3390/ani15213224

**Published:** 2025-11-06

**Authors:** María G. Martínez-Morales, Oscar E. Juárez, Dariel Tovar-Ramírez, Clara E. Galindo-Sánchez, Claudia Ventura-López, Carlos Rosas, Héctor Nolasco-Soria, Bertha Patricia Ceballos-Vázquez

**Affiliations:** 1 Centro Interdisciplinario de Ciencias Marinas, Instituto Politécnico Nacional, Av. IPN s/n, Col. Playa Palo de Santa Rita, La Paz 23096, Mexico; mguadalupemtzm@gmail.com; 2 SECIHTI—Estancias Posdoctorales por México, Centro de Investigaciones Biológicas del Noroeste, S.C., Av. IPN # 195, Col. Playa Palo de Santa Rita, La Paz 23096, Mexico; ojuarez.bio@gmail.com; 3 Centro de Investigaciones Biológicas del Noroeste, S.C., Av. IPN # 195, Col. Playa Palo de Santa Rita, La Paz 23096, Mexico; hnolasco04@cibnor.mx; 4 Centro de Investigación Científica y de Educación Superior de Ensenada, Baja California, Carretera Ensenada—Tijuana # 3918, Zona Playitas, Ensenada 22860, Mexico; clareliza@gmail.com (C.E.G.-S.); claudia.ventura.3010@gmail.com (C.V.-L.); 5 Unidad Multidisciplinaria de Docencia e Investigación, Facultad de Ciencias, Universidad Nacional Autónoma de México, Puerto de Abrigo S/N, Hunucmá 97356, Mexico; crv@ciencias.unam.mx

**Keywords:** venom gland, neuroendocrine regulation, metabolic specialization, transcriptomic analysis, digestion, antimicrobial peptides

## Abstract

Pacific pygmy octopus *Paroctopus digueti* is a promising model for cephalopod research and aquaculture; its feeding and nutritional biology remain poorly understood. The anterior salivary glands (ASG), posterior salivary glands (PSG), and digestive gland (DG) are central to these processes, but molecular comparisons are lacking. To address this gap, we performed a transcriptomic study to explore the enzymatic repertoire and functional specialization of these tissues. Total RNA was extracted from ASG, PSG, and DG of three pre-adult individuals collected in La Paz Bay, Mexico. RNA-Seq libraries were sequenced, and a non-redundant multi-tissue transcriptome was assembled. The ASG displayed high expression of neuropeptides, playing a role in neuroendocrine regulation. The PSG showed elevated protease expression, supporting its function in extracellular digestion, alongside toxins that reinforce its role as a venom gland. The DG was enriched in proteins linked to biomolecule catabolism and antimicrobial peptides, alluding to metabolic specialization and immune defense. These results were validated by qPCR, and target genes were also amplified in *Octopus maya* and *O. hubbsorum*, showing some similarities in expression patterns. Overall, our findings suggest strong glandular specialization in *P. digueti*, providing insights into cephalopod digestive physiology and supporting its value as a model species.

## Simple Summary

Pacific pygmy octopus *Paroctopus digueti* is a small species that could become an important model for octopus biology and aquaculture. To better understand how it captures and digests food, we studied three main organs that synthesize and secrete digestive enzymes and functional molecules in the feeding process: the anterior and posterior salivary glands and the digestive gland. We discovered clear differences in their functions by analyzing the genes active in each tissue. In the anterior salivary glands, gene expression was associated with signaling pathways that could regulate feeding processes. Gene expression in the posterior salivary glands was associated with the release of digestive enzymes and toxins that may help paralyze prey. This means they participate in the first digestion and act as a venom gland. Gene expression in the digestive gland suggests specialization in breaking down the food into molecules, producing energy reserves, and protecting the animal with antimicrobial molecules. These findings show that each gland has a unique role, helping us to understand how octopuses feed and defend themselves from pathogens. Furthermore, these results support *P. digueti* as a valuable species for future research and aquaculture development.

## 1. Introduction

The pigmy octopus, *Paroctopus digueti* (Perrier & Rochebrune, 1894), from the Mexican Pacific, is considered a promising candidate for aquaculture due to its body size, short life cycle (approximately eight months at 25 °C) without paralarval stage, high growth rate, and fecundity (up to 300 eggs per clutch) [1,2,3,4,5]. These features have enabled its successful conditioning in captivity and laboratory settings [1,6], positioning it as an emerging model species. This underlines the importance of deepening our understanding of its feeding biology and nutritional physiology.

Cephalopods are predators that employ sophisticated strategies for prey capture and digestion [7,8]. In these processes, the anterior salivary glands (ASG), posterior salivary glands (PSG), and the digestive gland (DG) play fundamental roles [9,10].

Although the ASG is relatively understudied in mollusks, it has been reported that they are composed of enterochromaffin cells (presumably serotonin-secreting) and salivary cells that release glycoprotein-rich secretions, which may promote chyme movement and enhance the action of PSG secretions [10,11]. Additionally, a proteomic analysis revealed the presence of chitinases, hyaluronidases, serine proteases, abundant cysteine-rich secretory proteins, antigen 5, and pathogenesis-related proteins (CAP proteins), and a high concentration of histones in the ASG, many of which are also found in the PSG [12]. The action of a mixture of bioactive compounds in the saliva, secreted by the paired ASG and PSG, enables effective prey immobilization and manipulation [7,12,13,14,15,16,17].

PSG secretions participate in predigestion of prey, enabling the disintegration of joints and muscle attachments [8,9,16], and in the extracellular digestion through the secretion of proteolytic enzymes such as chymotrypsin, trypsin, and carboxypeptidases [15]. This facilitates chyme formation and its transit through the crop, stomach, cecum, and eventually to the DG [9], where intracellular digestion occurs.

Beyond its role in extracellular digestion, the PSG—also known as the “venom gland” [10,18,19]—has been extensively studied, with particular focus on the characterization of venom components transported in the saliva [17,18,20,21,22]. Venom composition is species-specific, and this variation has been linked to feeding habits, geographic distribution, and ontogeny [18,21]. Commonly identified compounds include neurotoxins such as cephalotoxin and tachykinin, biogenic amines like serotonin and octopamine, and a variety of enzymes, including carboxypeptidases, chitinases, hyaluronidases, CAP proteins, and serine proteases [17,18,20,21]. A recent study also reported the presence of antimicrobial peptides (AMPs), suggesting that the PSG may contribute to immune defense [22].

The DG is a multifunctional organ responsible for enzyme secretion, intracellular digestion, nutrient absorption, excretion, and nutrient storage [9]. It is therefore considered a reliable indicator of the nutritional status of individuals [4]. Enzymatically, it produces most digestive enzymes involved in nutrient processing and mobilization. Given the protein-rich diet of octopuses, proteases such as chymotrypsin and trypsin are particularly relevant. However, other enzymes have also been identified, i.e., carbohydrases, lipases, acid and alkaline phosphatases, amylases, cathepsins, among others [4,23,24,25,26,27,28].

In cephalopod species, the feeding, digestion, and nutrition have been previously explored, and salivary and digestive glands have been studied from different perspectives as anatomical [9,13,14], histochemical [11,13], enzymatic [4,16,23,29,30,31,32], physiological [17,25,27,28,33,34,35], proteomic [12,21,22], and transcriptomic [19,21,24,36,37,38]. Notwithstanding, no molecular comparison among the three glands has been conducted to date. In this study, we analyzed the ASG, PSG, and DG transcriptomes in pre-adult *Paroctopus digueti* to compare their enzymatic repertoire and to describe the functional specialization of each gland. The results were verified by qPCR, and key genes were tested on two commercially important species.

## 2. Materials and Methods

### 2.1. Ethical Considerations

This research was conducted in accordance with the current legislation for the protection of the use of animals for scientific purposes, as outlined in the European Parliament Directive 2010/63/EU and the Mexican Government (NOM-062-ZOO-1999) for the production, care, and use of experimental animals [39]. Their anesthesia and humane euthanasia were performed according to previously established protocols [40,41,42], as was their handling [43].

### 2.2. Capture and Maintenance

Following the ‘3Rs Principle’ for animal research (Replacement, Reduction, Refinement) as well as ethical considerations for cephalopod research [40,43], we minimized the sample size (*n* = 3 per condition, as is common in standard RNA-seq analyses [44]). In this regard, three healthy pre-adults of *Paroctopus digueti* were captured by free diving at the Ensenada de La Paz, Baja California Sur, Mexico. They were placed in buckets with seawater and aeration, then transported to the Mariculture Pilot Unit of the Interdisciplinary Center of Marine Sciences (Centro Interdisciplinario de Ciencias Marinas del Instituto Politécnico Nacional, CICIMAR-IPN). The organisms presented an average total body weight of 19.8 g (2.7 SD), with an average dorsal mantle length of 3.4 cm (0.5 SD), and an average total length of 12.2 cm (0.3 SD) without visibly developed gonads, which corresponds to the pre-adult stage [45]. They were kept in a 200 L tank, connected to an open-flow system of mechanically and biologically filtered seawater and treated with ultraviolet (UV) light. They were individualized in 4” diameter polyvinyl chloride (PVC) and mesh shelters, provided with continuous aeration, and they were kept fasting until sampling, the next day.

### 2.3. Sampling

Organisms were anesthetized before dissection by immersion in cold water at 4 °C until relaxed [41] to enable a humane euthanasia procedure, which was performed through an incision between the eyes to achieve an immediate brain disconnection [40]. Dissection was performed using sterile materials, which were washed with chlorine (10%), ethanol (70%), and nuclease-free water (DEPC 0.1%) after each sample to avoid cross-contamination. The anterior salivary glands (ASG), posterior salivary glands (PSG), and digestive gland (DG) were obtained from each *P. digueti* pre-adult. To further validate the observed gene expression patterns also in other octopus species, the same sampling was carried out on pre-adult organisms (*n* = 3) of *Octopus maya* and *Octopus hubbsorum.* Samples were preserved in RNAlater (Thermo Fisher Scientific, Carlsbad, CA, USA) at 4 °C for 24 h and then stored at −80 °C in the Comparative Physiology and Functional Genomics laboratory of the Centro de Investigaciones Biológicas del Noroeste, S.C. (La Paz, Baja California Sur, Mexico).

### 2.4. RNA Extraction, Library Preparation, and Sequencing

Total RNA was extracted from glands of the three species using Trizol (Thermo Fisher Scientific, Carlsbad, CA, USA). Tissues were homogenized (20 mg approx. of each sample) using the Fastprep-24 TM 5G (M.P. Biomedicals, Santa Ana, CA, USA) instrument, with the aid of glass microbeads at a speed of 6.0 m/s for 40 s. Subsequently, RNA was isolated using chloroform and washed with 75% ethanol. RNA quality was assessed by electrophoresis on 1.5% agarose gels and quantified using a Nanodrop 2000 spectrophotometer (Thermo Fisher Scientific, Carlsbad, CA, USA).

The total RNA of each sample from *P. digueti* was precipitated with a mixture of 0.1 volumes of sodium acetate at 3M concentration and 3 volumes of absolute ethanol. RNA pellets were sent to the Functional Genomics of Marine Organisms Laboratory of the Centro de Investigación Científica y Educación Superior de Ensenada, Baja California (Ensenada, Baja California, Mexico), for the preparation of strand-specific RNA-Seq libraries. The libraries were synthesized from 1 μg of total RNA per sample. RNA concentration was measured with a Qubit 4 fluorometer (Thermo Fisher Scientific, Carlsbad, CA, USA), and integrity was assessed with the Bioanalyzer 2100 instrument (Agilent, Santa Clara, CA, USA). Library construction was performed with the Illumina Stranded mRNA Prep kit (Illumina, San Diego, CA, USA), following the manufacturer’s instructions. The libraries’ fragment size and concentration were verified with Bioanalyzer 2100 and Qubit, respectively. A total of 24 libraries were sequenced on the NovaSeq Plus platform (Illumina) from Novogene Corporation (Sacramento, CA, USA), generating approximately 20 million paired-end reads per library with a read length of 2 × 150 nt.

### 2.5. Bioinformatic Analysis

#### 2.5.1. Transcriptome Assembly

The quality of the raw sequencing reads was analyzed with FastQC v0.11.8 [46]. Subsequently, sequencing adapters and low-quality reads were discarded with Trimmomatic v0.39 using the following parameters (leading:5, trailing:5, slidingwindow:4:15, minlen:40) [47]. Clean reads were grouped according to tissue type (ASG, PSG, and DG). Additional libraries from the same glands of senescent organisms, and embryos in the organogenesis and growth phases, were retrieved from GenBank (SRR34402224-36, SRR34402242, and SRR34402243) and included in the transcriptome assembly, obtaining a more complete transcriptome assembly, but were not included in the posterior analysis. The assembly for each tissue type was performed using Trinity v2.15.2 with the stranded library mode and default parameters [48]. Four independent assemblies were obtained (embryo, ASG, PSG, and DG). Each assembly was filtered using TransRate v1.0.3 [49] by evaluating the alignment of original paired-end reads on the assembled contigs. The initials of the corresponding tissue were added to the contigs’ ID labels. Then, a multi-tissue assembly (including Embryo, ASG, PSG, and DG contigs) was integrated. Finally, to avoid transcript redundancy, CD-HIT v4.8.1 was implemented to select a representative contig from contig clusters (identity > 95%) [50], then all trinity-isoforms with an expression value below 20% of that of the dominant isoform were removed before downstream analysis. Coding sequences were identified, and gene products were predicted using TransDecoder v5.7.1 [51].

#### 2.5.2. Transcriptome Annotation

A custom database was created for transcriptome annotation. For this purpose, all cephalopod proteins (reviewed and unreviewed), all reviewed proteins of the phylum Mollusca, all unreviewed enzymes of the phylum Mollusca, and all proteins of venoms and toxins (ToxProt database) were downloaded from the UniProt Knowledgebase (https://www.uniprot.org/uniprotkb, accessed on 1 July 2024). Venom proteins and toxins were included to improve the annotation of the PSG, which is also the venom gland of the octopus [12,21]. In addition, considering the antimicrobial activity previously detected in cephalopod salivary glands, the antimicrobial peptide (AMP) database previously developed by Almeida et al. [52] was included. Annotation was performed by BLAST v2.2.29 searches [53] in our custom database (blastx for complete transcripts and blastp for predicted gene products). A minimum value of E-05 was accepted for BLAST results. To confirm the results, all putative toxin, venom, and AMP transcripts were reanalyzed by running BLASTn (megablast) online with the complete NCBI nucleotide database. Then, we kept only those transcripts showing hits with the same protein in both databases (ToxProt + NCBI for venoms and toxins, and AMP database + NCBI for AMPs). Finally, transcriptome completeness and integrity were assessed with BUSCO v5.8.1 using the mollusca_odb10 dataset [54].

#### 2.5.3. Differential Gene Expression Between Glands

To avoid biases when quantifying transcripts, duplicate paired-end reads from all pre-adult gland libraries were identified and removed using Nubeam-dedup [55]. Transcript abundance was estimated using Salmon v1.10.3 [56]. To assess dispersion between biological replicates, Pearson correlations and principal component analysis were performed using the Perl script “PtR” included in the Trinity v2.15.2 package [48]. Differential expression (DE) was estimated with the DESeq2 v1.42.0 package of R v4.4.3 [57], where an FDR of 0.001 and a fold change of |2| were set as significance thresholds. Transcripts with DE were clustered into groups with similar expression patterns using a hierarchical clustering tree subdivided at 70% of the total height [51]. Contig IDs were extracted from each subgroup (overexpressed genes in each tissue: ASG, PSG, and DG) for functional enrichment analyses.

#### 2.5.4. Functional Enrichment Analysis

Functional enrichment analyses were performed for differentially expressed genes (DEGs) based on gene ontology (GO) information retrieved from the UniProt database (August 2024). This analysis was carried out [51] using the R package Goseq v4.4.2 [58]. Genes associated with food digestion, biosynthesis, and signaling processes were identified from the enriched functional categories. For each tissue type, gene expression levels of selected DEGs were represented by heatmaps using the pheatmap package v1.0.13 [59] in RStudio v2025.05.0+496. Heatmaps were constructed based on the expression matrices normalized with edgeR v4.0.16 by the TMM method [60].

Finally, three transcripts (one per gland) showing tissue-specific expression were selected for validation by RT-qPCR.

### 2.6. Validation of Differential Expression via RT-qPCR

Validation was performed by real-time quantitative PCR (RT-qPCR) estimation of relative gene expression, also including *O. maya* and *O. hubbsorum* samples. RNA samples were treated with DNAase (Promega, Madison, WI, USA) from 2 µg of each sample, and cDNA synthesis was performed implementing the ImProm-II Reverse Transcription System kit protocol (Promega, Madison, WI, USA). As reference genes, the *elongation factor 1-beta*, the *proton ATPase type V subunit D*, and the *eukaryotic translation initiation factor 2A* were included. The candidate reference genes were selected considering their stability reported in previous works [61,62] and an in-silico evaluation of their expression in the RNA-Seq analysis (Figure S1). Primer pairs were designed for each transcript with Primer3web v4.1.0 (https://primer3.ut.ee/, accessed on 1 March 2025) [63]. The sequences of the primers used for the RT-qPCR validation analysis, their alignment temperatures, and efficiency are shown in Table 1.

The amplification efficiency of the primers was calculated following the Minimum Information for Quantification Experiments in Real Time (MIQE) publication guidelines [64], using a pooled cDNA sample prepared from the three biological replicates of each gland. Four stepwise dilutions (with a dilution factor 1:4) of this pooled cDNA in 0.01% DEPC-treated water were used to generate the standard curve. The qPCR was performed using a Bio-Rad CFX 96 thermocycler (Bio-Rad Laboratories, Hercules, CA, USA). Amplification reactions were performed in quadruplicate. The reaction consisted of 5 µL of SSoAdvanced Universal SYBR Green Super Mix (Bio-Rad Laboratories, Hercules, CA, USA), 0.3 µL of forward and reverse primers at a concentration of 10 mM, 3.4 µL of 0.01% DEPC water, and 1 µL of sample cDNA (1:4 dilution, equivalent to 12.5 ng total RNA), for a final volume of 10 µL. The temperature program consisted of 30 s at 95 °C for initial polymerase activation, 40 cycles starting at 95 °C for 5 s for DNA denaturation, and 30 s at 59 °C for primer alignment, amplicon extension, and plate readout. A melting curve analysis, with gradual increase from 65 °C to 95 °C with 0.5 °C increments every 5 s, was included to corroborate the specificity of the PCR products.

The stability of reference gene expression was evaluated using the online version of the RefFinder program (https://www.ciidirsinaloa.com.mx/RefFinder-master/, accessed on 1 July 2025) [65], which integrates the geNorm [66], NormFinder [67], and Delta-Ct [68] methods. Relative expression of target genes was estimated using the Delta Cq method with efficiency correction [69]. Since relative expression values did not meet the assumptions of normality (Shapiro–Wilk) and homoscedasticity (Levene) [70], the Kruskal–Wallis nonparametric test, followed by Dunn’s post hoc test [71], was implemented to compare gene expression among glands from *P. digueti*. However, in the comparison among species, no deviation from normality was detected; therefore, an ANOVA test, followed by Tukey’s HSD parametric test, was implemented. For all tests, a significance level α of 0.05 was used. Statistics were calculated in RStudio version 2025.05.0+496 [72].

## 3. Results

### 3.1. Transcriptome Assembly and Annotation

For each tissue (embryo, ASG, PSG, and DG), an average of 150,469,322 raw paired-end reads was obtained, totaling 601,877,291. These raw sequencing data were deposited in the NCBI SRA database (accession number: SRR34402220-SRR34402243). After quality filtering and removal of duplicated read pairs, the average paired-end reads per tissue was 79,863,243, with a total of 319,452,973 read pairs used for the construction of the multi-tissue transcriptome.

The multi-tissue partial transcriptome of *Paroctopus digueti* presented a total of 891,337 high-quality reconstructed transcripts, corresponding to 865,931 genes with an N50 of 469 nt. The number of annotated transcripts was 216,640, corresponding to an annotation rate of 24.3%. However, coding sequences (79,265) showed a higher annotation rate (65.7%) than total transcripts. The assembly was deposited in the NCBI TSA database (accession number: GLHK000000000000). According to the BUSCO analysis, this partial transcriptome of *P. digueti* includes 90.4% of molluscan orthologous genes, and only 9.6% are missing. From the present orthologous genes, 54.1% are complete and single-copy, 31.2% are complete and duplicated, and 4.2% are fragmented.

Eighteen contigs with significant similarity to proteins from the venom and toxin database (ToxProt) were identified in *P. digueti* glands. Identity values ranged from 78% to 100%, indicating a high similarity to known venom proteins, while E-values ranged from 0 to 1 × 10^−104^, supporting a strong probability of protein homology. Among the identified transcripts, we find the Acetylcholinesterase, encoding a neurotoxic enzyme, that was detected in both salivary glands. Venom components such as Hyaluronidase-1, Venom dipeptidyl peptidase 4, and Venom phosphodiesterase were also detected. Additionally, transcripts related to cardiovascular regulation were identified, including a putative Tachykinin-2 present only in PSG, and Neprilysin-1 and Plancitoxin-1 detected in ASG and DG (Table A1).

Furthermore, 34 contigs showed significant homology to proteins in the antimicrobial peptide (AMP) database [52]. Identity values ranged from 66% to 99%, and E-values ranged from 0 to 3 × 10^−30^, suggesting protein homology. Transcripts with the highest homology, included ubiquitins (absent in the PSG) with identity percentages greater than 85%, as well as multiple histones, including H2A, H2B, and H4, with identity percentages greater than 91%. Lysozyme-like proteins (Lysozyme, Lysozyme1, and Lysozyme3) were also identified in the three glands, with identity percentages ranging from 83 to 98%.

**Figure 1:**
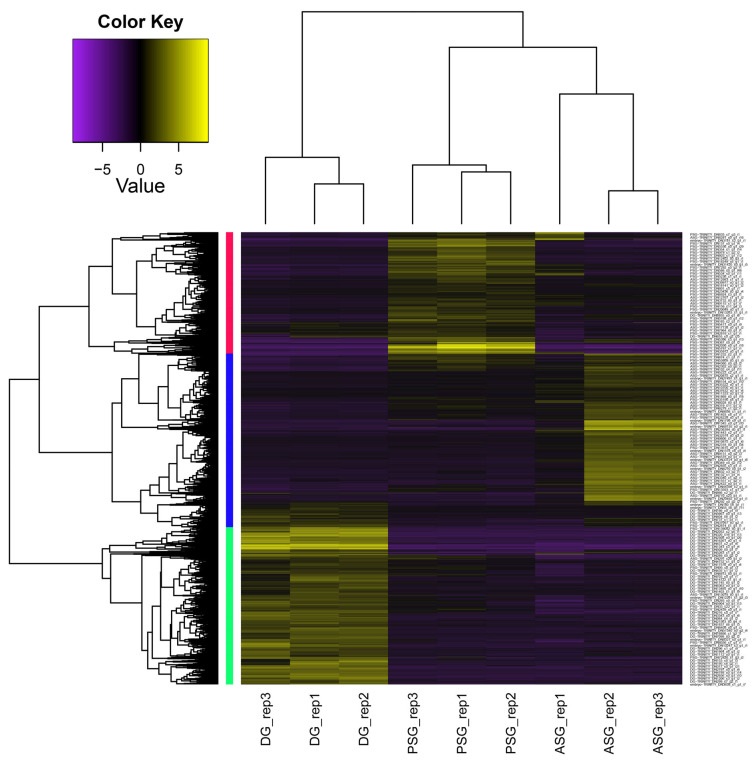
Heatmap of DEGs including a hierarchical clustering tree. Expression patterns in three glandular tissues digestive gland, posterior salivary glands, and anterior salivary glands (DG, PSG, and ASG) of the digestive tract of *Paroctopus digueti*, each with three biological replicates, are shown. Numerical values represent Z-scores of gene expression. The color scale indicates the intensity of expression: purple = underexpression, black = moderate expression, and yellow = overexpression. The dendrograms reflect similarity between samples (top), and between transcripts (left), evidencing that the main expression clusters (red, blue, and green) correspond to upregulated genes in a particular gland.

The hierarchical clustering tree (Figure 1) demonstrated coherent clustering of biological replicates from each tissue, thereby suggesting that each gland possesses a distinct and well-defined transcriptomic profile. Furthermore, the tree indicates a higher degree of similarity between ASG and PSG, with DG being the most divergent.

The subgroup with the highest expression in ASG has 3701 transcripts, the subgroup with the highest expression in DG has 3360 transcripts, and the subgroup with the highest expression in PSG has 2590 transcripts.

### 3.2. Functional Enrichment and DEG’s

**Figure 2:**
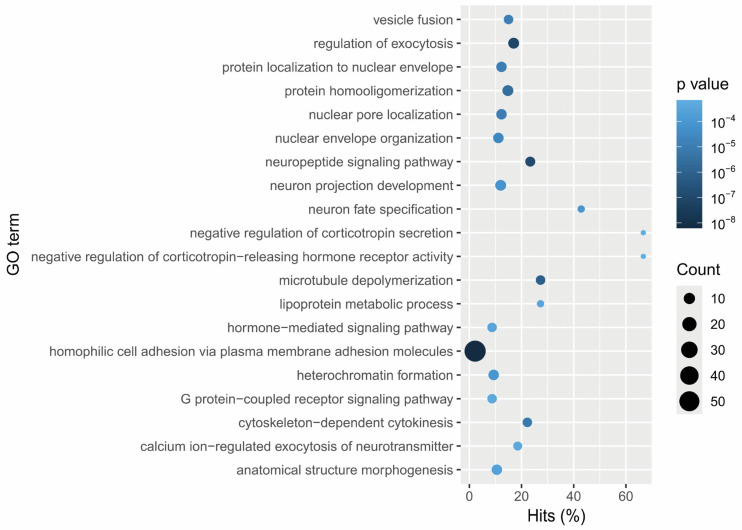
Enrichment of GO terms (biological processes) in the Anterior Salivary Gland of *Paroctopus digueti*. The bubble plot shows the major biological processes enriched for the upregulated transcripts in the ASG. The *Y*-axis lists the GO terms, while the *X*-axis indicates the percentage of genes detected in the tissue for each category (Hits%). The size of each circle represents the number of genes annotated under that GO term (Count), and the color gradient indicates the level of statistical significance (*p*-value) with darker tones representing higher significance. Among the most relevant processes detected, we highlight the regulation of corticotropin secretion, neuropeptide signaling, and hormone-mediated signaling pathways.

Functional enrichment analyses were performed based on gene ontology (GO) terms, and we focused on those terms corresponding to biological processes (BP). In the ASG (Figure 2), we detected significant representation of genes related to vesicular fusion, regulation of exocytosis, neural projection, and neuropeptide signaling, among others. Additionally, the terms with the highest hit percentage (% of genes from a specific category) were the negative regulation of corticotropin secretion and neural fate specification. The term with the highest count (no. of associated genes) was the homophilic cell adhesion. The enriched categories for the ASG included genes like Synaptotagmin-11, Neuropeptide prohormone-4, Neuroendocrine protein 7B2, and FMRFamide neuropeptide, indicating that the ASG plays a role in neuroendocrine regulation.

**Figure 3:**
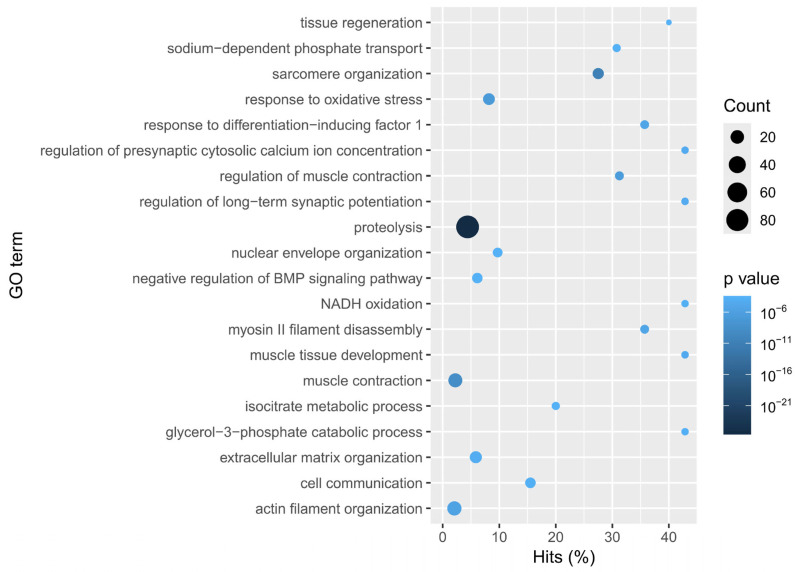
Enrichment of GO terms (biological processes) in the Posterior Salivary Gland of *Paroctopus digueti.* The bubble plot shows the major biological processes enriched for the upregulated transcripts in the PSG. The *Y*-axis lists the GO terms, while the *X*-axis indicates the percentage of genes detected in the tissue for each category (Hits%). The size of each circle represents the number of genes annotated under that GO term (Count), and the color gradient indicates the level of statistical significance (*p*-value) with darker tones representing higher significance. Relevant enriched terms include tissue regeneration, oxidative stress response, and proteolysis.

Functional enrichment analysis in the PSG (Figure 3) revealed significant representation of genes related to oxidative stress responses, muscle contraction, and proteolysis, which also had the highest counts. Additionally, processes such as synaptic and presynaptic regulation, glycerol-3-phosphate catabolism, and NADH oxidation had the highest proportions of hits. These enriched categories included genes like Trypsin alpha-3, S1 type peptidase, and Chymotrypsin-1, supporting its importance in extracellular digestion.

**Figure 4:**
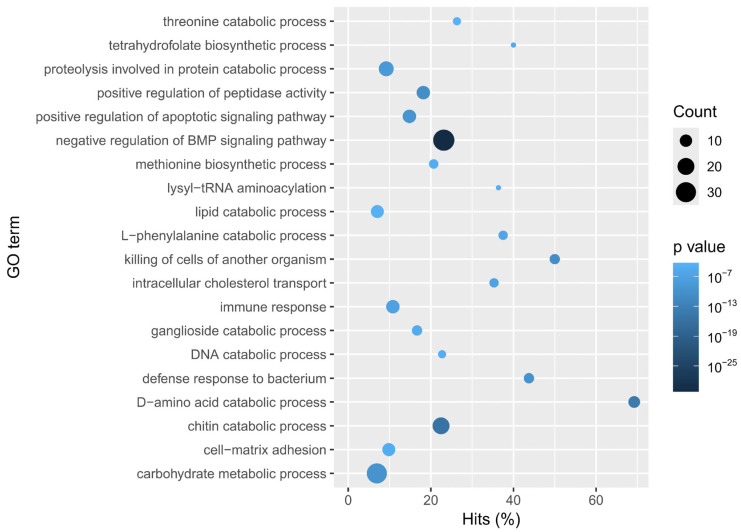
Enrichment of GO terms (biological processes) in the Digestive Gland of *Paroctopus digueti*. The bubble plot shows the main biological processes enriched for transcripts upregulated in the DG. The *Y*-axis lists the GO terms, while the *X*-axis indicates the percentage of genes detected in the tissue by each category (Hits%). The size of each circle represents the number of genes annotated under that GO term (Count), and the color gradient indicates the level of statistical significance (*p*-value) with darker tones representing higher significance. Relevant terms include lipid catabolism, D-amino acid catabolism, and immune response.

**Figure 5:**
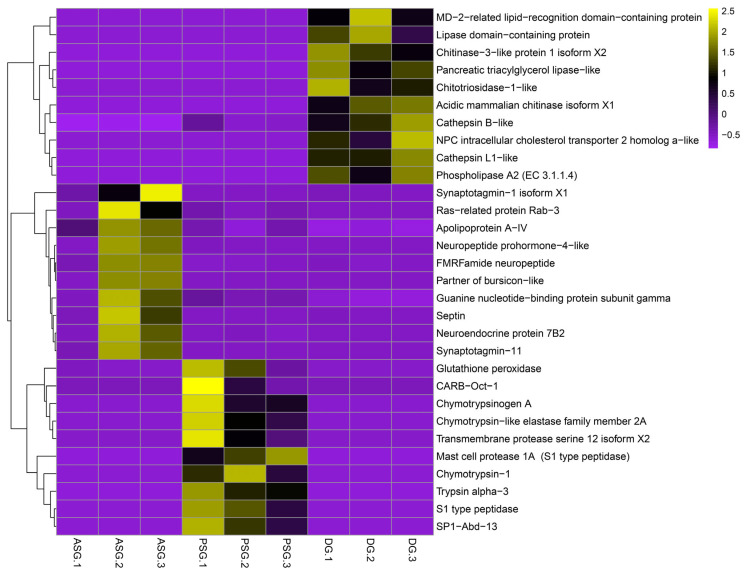
Heatmap of tissue-specific genes of *Paroctopus digueti*. Clustering by expression of the identified DEGs (*Y*-axis) in each tissue (ASG, PSG, and DG, *X*-axis) is shown. Z-scores of normalized gene expression are represented on a color scale ranging from −1 (purple = underexpression) to 2.5 (yellow = overexpression), with 0 (black) indicating moderate or average expression. Hierarchical clustering was applied to transcripts and samples, revealing tissue-specific expression profiles.

Overexpressed genes of DG enriched catabolic processes of amino acids, proteins, lipids, DNA, and chitin. The terms with a higher number of genes associated were the BMP negative regulation, carbohydrate metabolism, and chitin catabolism. On the other hand, D-amino acid catabolism had the highest hit percentage (Figure 4). Enriched categories included genes like Chitinase-3, Cathepsin B and L1, and the NPC intracellular cholesterol transporter 2, confirming its specialization in intracellular digestion and nutrient metabolism. The expression values of DEGs associated with the most relevant biological processes (highest functional enrichment) were plotted as heatmaps (Figure 5).

**Figure 6:**
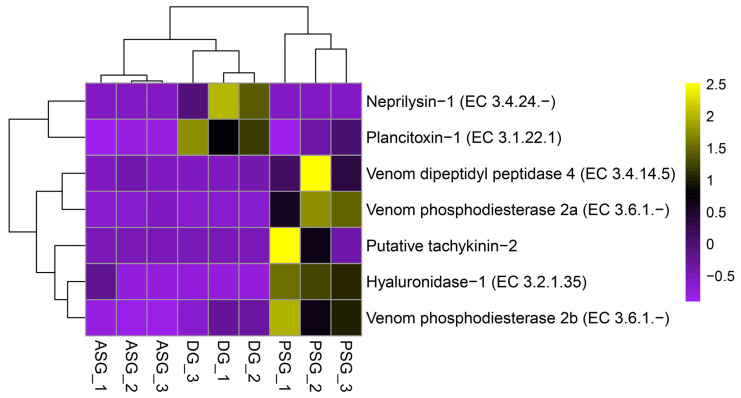
Heatmap of differentially expressed toxin and venom transcripts of *Paroctopus digueti*. Clustering by expression of toxin and venom genes (*Y*-axis) with differential expressions among tissues (ASG, PSG, and DG, *X*-axis). Z-scores of normalized gene expression are represented on a color scale ranging from −1 (purple = underexpression) to 2.5 (yellow = overexpression), with 0 (black) indicating moderate or average expression. Hierarchical clustering was applied to transcripts and samples, revealing tissue-specific expression profiles.

Regarding the expression of toxins and venoms, no transcript showed elevated expression in ASG. The PSG, however, showed high expression of transcripts encoding venom protein homologs, such as two Venom phosphodiesterase isoforms, a putative Tachykinin-2, Hyaluronidase-1, and Venom dipeptidyl peptidase 4. This supports its role as the venom gland in *P. digueti*. The DG also showed high expression of transcripts encoding homologs of the Neprilysin-1 and Plantitoxin, a toxin described in a starfish [73] (Figure 6).

**Figure 7:**
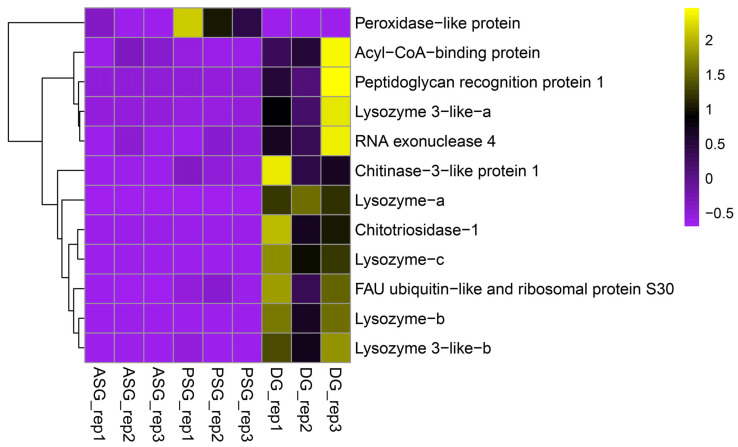
Heatmap of differentially expressed antimicrobial peptide (AMP) genes of *Paroctopus digueti*. Clustering by expression of the identified antimicrobial peptide genes (*Y*-axis) with differential expression among tissues (ASG, PSG, and DG, *X*-axis). Z-scores of normalized gene expression are represented on a color scale ranging from −1 (purple = underexpression) to 2.5 (yellow = overexpression), with 0 (black) indicating moderate or average expression. Hierarchical clustering was applied to transcripts and samples, revealing tissue-specific expression profiles.

Concerning the AMP genes, DG stood out, showing the highest expression in eleven of the twelve differentially expressed AMPs detected among the glands, including the acyl-CoA-binding protein, five lysozyme variants, Peptidoglycan recognition protein 1, RNA exonuclease 4, Chitinase-3, Chitotriosidase-1, and FAU ubiquitin. The PSG only showed higher expression of a Peroxidase-like protein. We did not detect any AMP genes showing higher expression in the ASG (Figure 7).

### 3.3. Validation of Differential Gene Expression

To validate tissue-specific expression, we selected the neuropeptide gene *FMRFamide*, which is highly expressed in ASG; the gene encoding Chymotrypsin-1, which is highly expressed in PSG; and the gene for the Intracellular cholesterol transport protein NPC2, which is highly expressed in DG. This selection was based on their RNA-Seq expression patterns and functional relevance. As the reference gene, the Proton ATPase V-type D-subunit was selected, besides being the only reference gene to amplify in the three species, its expression was the most stable among the different tissues. It is worth mentioning that the primers for Eukaryotic initiation factor 2A and the Elongation factor 1B were not efficient in *O. hubbsorum*.

**Figure 8:**
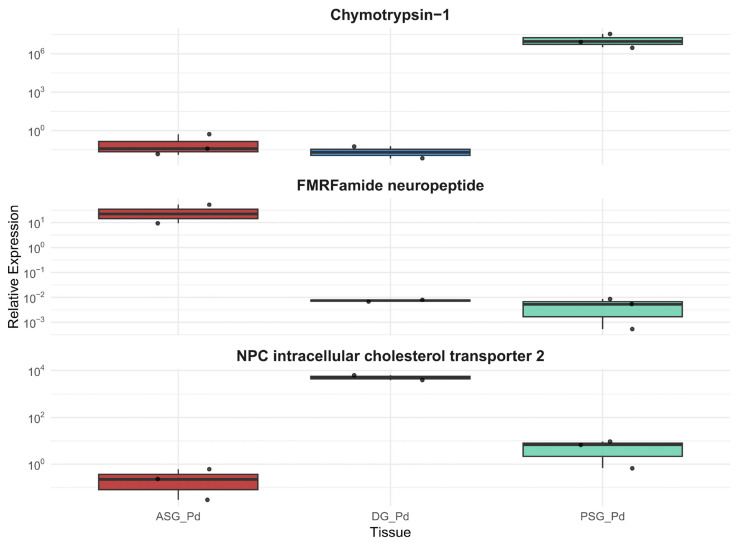
Comparison of relative expression by RT-qPCR of selected ASG, PSG, and DG genes in *Paroctopus digueti*. Each panel shows the expression of a tissue-specific gene, encoding the proteins: Chymotrypsin-1, FMRFamide neuropeptide, and NPC intracellular cholesterol transporter 2. Relative expression values were obtained using the Proton ATPase V-type subunit-D as a reference gene. Data is represented in box plots, indicating each tissue’s median, quartiles, and outliers. Tissue-specific gene expression coincides with RNA-Seq results.

Expression of the gene coding for the enzyme Chymotrypsin-1 showed significant differences between tissues (*p* < 0.05), with higher expression in PSG. The gene encoding the neuropeptide FMRFamide was exclusively expressed in the ASG, and the gene for the Intracellular cholesterol transport protein NPC 2 showed significant differences between tissues (*p* < 0.05), with higher expression in the DG (Figure 8). These results were in complete agreement with those obtained via RNA-Seq.

**Figure 9:**
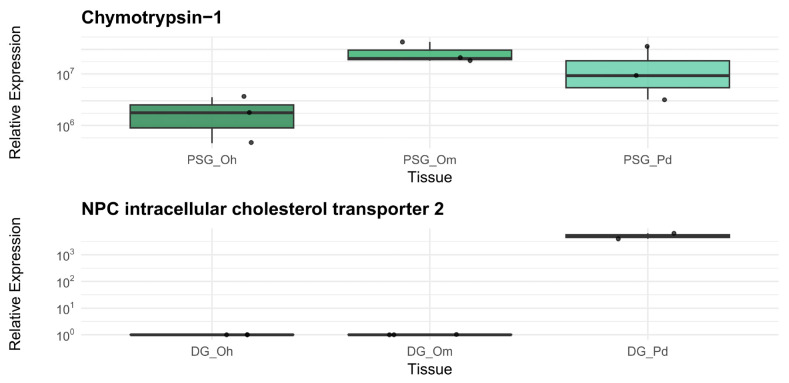
Comparison of relative expression by RT-qPCR of selected PSG and DG genes in three octopus species. Each panel shows the expression of a gene in a specific gland, encoding the proteins: Chymotrypsin-1 (top) in the PSG and Intracellular NPC cholesterol transporter 2 (bottom) in the DG. In both cases, the expressions were compared in tissues from *Paroctopus digueti* (Pd), *Octopus maya* (Om), and *O. hubbsorum* (Oh). Relative expression values were obtained using the Proton ATPase V-type subunit-D as a reference gene. Data is represented in box plots, indicating each tissue’s median, quartiles, and outliers. A similar expression of the gene encoding Chymotrypsin-1 is observed in the three species, unlike the gene encoding the NPC cholesterol transporter 2, which was amplified only in *P. digueti*.

Furthermore, amplification of the gene encoding Chymotrypsin-1 was successful in the PSG of the three octopus species, using the same primer pairs. The expression of this gene was similar in the three different species according to the ANOVA test, which showed no significant differences (*p* = 0.126; Figure 9). However, *O. maya* exhibited the highest expression values, followed by *P. digueti* and *O. hubbsorum*.

The expression of the gene encoding the Intracellular cholesterol transporter NPC 2 was significantly higher in the DG of *P. digueti* (*p* = 0.001; Figure 9) than in the other two species, where the gene was also amplified with the same primer pairs used for *P. digueti*, but its expression was close to zero.

Amplification of the gene encoding the FMRFamide neuropeptide was only successful in the ASG of *P. digueti*. No gene amplification was accomplished for the ASG of *O. maya* and *O. hubbsorum* (including target and reference genes).

## 4. Discussion

This work constitutes the first comparative transcriptomic analysis among the digestive tract glands of cephalopods, using the Pacific pygmy octopus *Paroctopus digueti* as a model.

The comparison was based on a multi-tissue partial transcriptome (around 90% complete) comprising 891,337 high-quality reconstructed transcripts. This value ranks among the most complete transcriptomes reported for cephalopods, considering only those evaluations using the Mollusca lineage dataset of BUSCO [42,74,75], and it is comparable to that obtained for *Octopus vulgaris* from embryo and paralarvae assemblies (87.2%) [42].

Of the complete genes, 31.2% were identified as duplicates. This redundancy is due to the inclusion of different tissues and developmental stages in the assembly, which contribute with multiple tissue-specific and stage-specific isoforms. A similar pattern was observed in *O. vulgaris*, albeit to a greater extent, with a duplication rate of 50.4% [42]. The fragmentation level of orthologous genes (4.2%) was relatively low considering that de novo assemblies typically generate several fragmented transcripts.

It is important to note that, to date, only PSG has been explored through both transcriptomic and proteomic approaches [12,21]. Therefore, the relatively low annotation rate (around 24%) observed in this study can likely be attributed to the scarcity of available molecular data for other digestive tract glands. In particular, no transcriptomic references exist for the ASG, which explains the high proportion of novel and unique transcripts identified in our dataset.

In general, well-defined transcriptomic profiles for each gland could be detected. The genes with the highest expression in the ASG were associated with neuroendocrine functions that could regulate feeding; genes with an expression peak in the PSG were associated with extracellular digestion and prey paralysis; and those genes conspicuous in the DG were associated with a highly specialized intracellular digestion. Likewise, the role of the PSG as a venom gland was supported. Furthermore, antimicrobial peptides (AMPs) detected in the DG points to an important participation of this gland in the immune response of *P. digueti*.

### 4.1. Anterior Salivary Glands

To our knowledge, this is the first RNA-Seq transcriptomic analysis for the ASG of any octopus species. Transcripts overexpressed in the ASG enriched processes related to neuropeptide signaling, cellular communication, nerve signaling, and synaptic transmission, including GO terms like *vesicle transport and release*, *vesicle fusion*, *regulation of exocytosis*, and *calcium-dependent neurotransmitter release*. These findings are consistent with the presence of enterochromaffin cells previously described in the ASG of cuttlefish, sepioles, and octopuses [11], which are also present in the gullet, stomach, and intestine of octopods [76,77]. Such cells communicate digestive system components with neural elements, transmitting environmental, immune, and vascular information, mainly through serotonergic pathways mediated by voltage-dependent calcium channels [78]. Among the genes included in the enriched categories, we found Synaptotagmin-1 and -11, associated with vesicular release and recycling [20,21], Neuropeptide prohormone-4, a neuropeptide precursor conserved in mollusks [22], and Neuroendocrine protein 7B2, a chaperone involved in the maturation of functional neuropeptides via the Prohormone convertase PC2 [23].

Another relevant category was the negative regulation of corticotropin secretion and its receptor. In *Octopus vulgaris*, the Corticotropin-releasing factor (CRF) has been reported in the optic lobe and peduncular complex, where it has been proposed to be involved in olfactory processing and in the integration of chemoreceptor and visuomotor functions [79]. In vertebrates, CRF is associated with appetite inhibition [80]. This opens a promising line of research into the potential involvement of ASG in prey selection and appetite regulation. In addition, CRF-releasing neurons may be immunoreactive to the neuropeptide FMRFamide [81].

In this regard, the neuropeptide FMRFamide, widely studied in cephalopods, showed the highest expression in the ASG of *P. digueti*. This neuromodulator is involved in heart rate control, reproduction, chromatophore regulation, and the modulation of feeding behavior and appetite [81,82,83,84,85,86]. Given its importance, FMRFamide was selected as a tissue-specific gene to validate RNA-seq results by RT-qPCR.

Although FMRFamide had been reported in the nervous system of *Sepia officinalis* [87] and in multiple neural and reproductive tissues of various octopus species [88,89,90,91] this study constitutes the first report of its detection in a digestive tract gland of cephalopods, specifically in the ASG of *P. digueti*, where it was validated via RT-qPCR. Unfortunately, no gene amplification (including target and reference genes) was accomplished in the ASG of *O. maya* and *O. hubbsorum*, probably due to metabolites produced by the gland that interfered with cDNA synthesis and PCR. Thus, *P. digueti* could be proposed as the most suitable octopus model for the transcriptomic study of the ASG.

Additionally, at least four transcripts encoding putative toxins were identified in ASG (for more details, see Appendix A). Among them, we found the transcript for Acetylcholinesterase, a glycoprotein linked to neurotoxin effects such as prey paralysis in snake envenomation [92]. Other toxins identified included the metalloprotease Neprilysin-1, and the hepatotoxin Plancitoxin-1, previously reported in cnidarians, and echinoderms [93,94], as well as the Venom dipeptidyl peptidase 4, which is also shared with PSG. Although these transcripts showed significantly higher expression in the PSG, these findings suggest that ASG of *P. digueti* may contribute to the secretion of toxic compounds that induce prey paralysis.

Regarding antimicrobial peptides (AMPs), ten transcripts were identified in ASG, including Chitinase-3 and several histone variants, in agreement with Fingerhut et al. [12]. However, AMPs showed lower expression in the ASG than in PSG and DG.

### 4.2. Posterior Salivary Glands

According to the Gene Ontology (GO) enrichment, transcripts overexpressed in PSG suggest key roles in muscle function, regeneration, stress response, and metabolism. Enriched GO terms were related to muscle fiber structure and dynamics, tissue formation and repair after damage or growth, as well as regulation of neural and neuromuscular signaling. These findings indicate the participation of PSG in predatory and feeding behaviors at the muscular level, functions that had not been previously reported in these glands, and that deserve to be explored with complementary techniques.

Metabolic functions associated with extracellular digestion and maintenance of sustained muscle activity were also observed, including GO terms like *proteolysis*, *NADH oxidation*, *glycerol-3-phosphate catabolism*, and *isocitrate metabolism*. Among the genes from the enriched categories, key proteases were identified, such as Trypsin Alpha-3 and Chymotrypsin-1 and 2A, widely studied in cephalopods and essential for the digestion of carnivorous diets [9,25,26,95,96]. The presence of these enzymes in PSG has been documented in *Eledone cirrhosa* [16], *Octopus sinensis* [30], *O. vulgaris* [95], *O. mimus* [33] and *O. maya* [97], showing higher proteolytic activity in the PSG versus the DG [25,95,98].

The activation of Chymotrypsinogen A in *O. vulgaris* PSG has also been reported [22,30], probably mediated by the arrival of first chyme [27] or by limited proteolysis by trypsin. The relationship between both trypsin and chymotrypsin enzymes, and their variation in different conditions, has been studied previously [25,97]. Although it is a topic of interest, we will delve into it in further work.

Chymotrypsin-1 was used as a tissue-specific marker for PSG to validate RNA-seq results by qPCR. Validation was successful for these genes, showing significantly higher expression in the PSG versus the ASG and the DG in *P. digueti*. The amplification of this gene with the same primer pairs in *O. maya* and *O. hubbsorum* was also successful, suggesting that this gene is evolutionarily conserved among octopus species. Moreover, no significant differences were detected between pre-adults of the three species, confirming the high protein demand common to all three [99].

Regarding the role of the PSG in toxin production, a total of eight genes were identified, five of which [12,19,21,38,100,101] were differentially expressed among the digestive tract glands and showed higher expression in PSG. These include the putative Tachykinin-2, a vasoactive neurotoxin reported in several octopus species [12,19,21,38,102], the allergen Venom dipeptidyl peptidase 4 and Hyaluronidase-1, involved in venom dispersal [12,21,22]. Notably, in contrast to *O. vulgaris*, where a higher proportion of hyaluronidases was observed in ASG, in *P. digueti*, this kind of enzyme showed higher expression in the PSG than in the ASG.

Moreover, two isoforms of Venom Phosphodiesterase 2 were identified; these toxins are common among snakes of the genus *Crotalus* [103], although their specific function in cephalopods remains uncertain. These findings further support the role of PSG as the main venom gland in *P. digueti.* Finally, the high expression in PSG of a transcript encoding a Peroxidase-like protein suggests that this gland also participates in antibacterial defense, coinciding with Almeida et al. [22].

### 4.3. Digestive Gland

Numerous authors have studied the DG of cephalopods, describing their cells, enzymatic secretions, and nutrient storage; however, there is no characterization at the transcriptomic level with an emphasis on the digestive process. Functional enrichment of genes with higher expression in the DG reflects a strong involvement in metabolism and degradation of biomolecules, along with cellular regulation and immune response mechanisms. Catabolic and biosynthetic pathways specific for amino acids—threonine, methionine, phenylalanine, D-amino acids—were predominant among the GO-enriched categories, which is consistent with the high growth rates in cephalopods and thus the high requirement for protein synthesis [104]. In addition, catabolic pathways of carbohydrates and complex components such as gangliosides and chitin, as well as controlled degradation of proteins and DNA, were significantly enriched for genes overexpressed in the DG.

GO terms related to lipid metabolism and transport were also enriched in the DG. In this regard, Mancuso et al. [95] found higher levels of lipase in the DG of *O. vulgaris*, compared to other tissues, including the PSG, and García-Garrido et al. [105] pointed out that lipids are important nutrients for cephalopods, mainly as a source of energy. This has been proven even in newborn juveniles of *P. digueti*, which depend on lipid reserves for survival [4]. On the other hand, genes involved in lipid transport were also highly expressed in the DG, particularly in the intracellular movement of cholesterol, which is key for membrane organization and signaling. In this category, we found the gene encoding the NPC intracellular cholesterol transporter 2, which was selected as a tissue-specific gene for qPCR validation of RNA-seq results in DG.

The qPCR analyses showed a significantly higher relative expression of the cholesterol transporter in the DG compared to PSG and ASG of *P. digueti*, validating the RNA-seq results. When the same primer was tested in *O. maya* and *O. hubbsorum*, the relative expression of this gene was close to zero for both species. Therefore, relative expression was significantly higher in *P. digueti*. Since several studies have documented the importance of lipid transport in octopuses of commercial interest, such as *O. vulgaris*, *O. maya*, and *O. mimus* [27,95], it is likely that the lower expression of this gene detected in *O. hubbsorum* and *O. maya* could be related to a reduced primer efficiency on both species, and/or the use of a specific isoform by each species. So further analysis will be required to answer the emerging questions.

Among the overexpressed genes found in DG, the presence of cathepsins B and L1 also stands out. These have recently been reported in *O. maya*, where they participate not only in protein hydrolysis but also as regulators of their enzymatic activity, adapting to physiological and environmental conditions and ensuring optimal digestion and survival [23,24]. However, the functions of these acid enzymes are still poorly studied.

On the other hand, the significant enrichment of GO terms related to pathogen defense and immune response stands out. This is congruent with the identification of genes encoding antimicrobial peptides (AMPs) in the DG, via BLAST alignment on the AMPs database of Almeida et al. [22]. According to gene expression values, the DG presented higher expression of lysozymes, ubiquitins, and the Chitinase-3, all with antimicrobial functions [22]. The Chitinase-3 is ubiquitously found in the saliva and salivary glands of cephalopods [21,38]. However, we detected transcripts of this gene in the three glands. Interestingly, the DG of *P. digueti* presented the highest expression, so it is possible that the most significant secretion of Chitinase-3 occurs in the DG and subsequently it flows through the tract, as is noted with other digestive enzymes [9,27].

Simply, chitinases’ function is to degrade chitin [12], which is abundant in the crustacean exoskeleton. However, they are also associated with external digestion of prey and infection of the host by bacteria, which causes damage to the prey and facilitates poisoning [18,21].

Six homologous transcripts encoding toxins and venom proteins were found in DG, of which two presented higher expression in this gland. The metalloprotease Neprilysin-1 and the hepatotoxin Plancitoxin-1, previously found in the ASG of marine organisms [93,94].

### 4.4. Functional Complementarity Among the Digestive Tract Glands

Functional enrichment analysis showed that the digestive tract glands cooperate in multiple biological processes [10,13]. In some cases, the same GO term was enriched in different glands, for example: *nuclesar envelope organization*, *cell communication*, *cell–matrix adhesion*, and *negative regulation of BMP signaling*. Moreover, enriched GO terms in the ASG like *neuropeptide signaling* and *hormone-mediated signaling pathway,* could be complementary to those enriched in the PSG associated with neuromuscular regulation (*presynaptic calcium regulation*, *muscle contraction*, *long-term synaptic potentiation*). Similarly, GO terms enriched in the PSG related to the energetic metabolism (*NADH oxidation*, *isocitrate metabolic process*) are linked to the catabolism of biomolecules (amino acids, lipids, carbohydrates, proteins, and DNA) in the DG.

These patterns suggest a functionally integrated system: the ASG acts as a neuronal signaling center in response to stimuli, the PSG participates in prey capture and immobilization, pre-digestion and extracellular digestion, and the DG transforms nutrients into energetic molecules and reserves necessary to sustain the metabolic demands and maintain homeostasis [27].

Although this study offers valuable insights into these processes, it should be emphasized that transcript detection alone does not confirm protein expression or biological activity, and additional experimental evidence is required to fully elucidate their functional implications (for example, evaluating specific enzymatic activities).

## 5. Conclusions

In summary, while the efficiency of the cephalopod digestive system has traditionally been attributed to the digestive gland, our results highlight the essential roles of the anterior and posterior salivary glands and reveal the functional specialization of each tissue. Transcriptomic evidence indicates that the ASG is mainly linked to signaling pathways regulating feeding, the PSG to extracellular digestion and prey paralysis, and the DG to specialized intracellular digestion and immune defense through antimicrobial peptides. Altogether, these findings not only expand the understanding of digestive physiology in octopuses but also support the suitability of *Paroctopus digueti* as a promising model species for cephalopod research, given its favorable biological traits for captive maintenance, such as small size, the absence of a paralarval stage, and a short life cycle.

## Figures and Tables

**Table 1:** Primers were used in RT-qPCR analysis to validate gene expression.

Transcript ID	Putative Protein		Tm	Sequence	Ps	E%
ASG-TRINITY_DN652_c0_g1_i9	FMRFamide neuropeptide	F	58.19	GGAACCTGACAAGCGATTCA	144	100.3
		R	59.75	TCTTCTTCCCCATTGCCTCG		
PSG-TRINITY_DN383_c0_g1_i1	Chymotrypsin-1	F	59.68	GATGGGGTGATCTCGGATGG	134	98.7
		R	59.07	GTCACCTGCACACAAGACG		
DG-TRINITY_DN585_c2_g2_i7	NPC intracellular cholesterol transporter 2	F	58.3	GGTCTCACTTGTCCCTTAAGC	149	99.5
		R	58.09	GCTGGGATCTGGACACAAAA		
PSG-TRINITY_DN3257_c3_g1_i1	ATPase subunit D	F	58.4	CCGAAGCCAAGTTTACCACA	147	99.8
		R	58.88	TGGTGTCTGTTCCGTCTTGA		
ASG-TRINITY_DN15872_c0_g1_i1	Elongation factor 1-beta	F	59.01	TGCCCTCATTGCCAAGAGTA	123	115
		R	59.46	GAAGCACCCCAGAGTAGACC		
Embryo-TRINITY_DN11489_c0_g1_i4	Eukaryotic translation initiation factor 2A	F	58.68	GCTGGCACTGGTTTACTTGT	149	105.7
		R	59.3	TGCCACTCCACACAATAGACA		

Terms: F = forward; R = reverse; Tm = melting temperature; Ps = product size; E = primer efficiency.

## Data Availability

The original contributions presented in this study are included in the article/Supplementary Materials. Further inquiries can be directed to the corresponding authors.

## Supplementary Materials

The following supporting information can be downloaded at: https://www.mdpi.com/article/10.3390/ani15213224/s1, Figure S1: Candidate Reference Genes.

## Abbreviations

The following abbreviations are used in this manuscript:

ASG	Anterior salivary glands
PSG	Posterior salivary glands
DG	Digestive gland
CAP	Cysteine-rich secretory proteins, Antigen 5, and Pathogenesis-related proteins
AMPs	Antimicrobial peptides
CICIMAR-IPN	Centro Interdisciplinario de Ciencias Marinas del Instituto Politécnico Nacional
UV	Ultraviolet
PVC	Polyvinyl chloride
DEPC	Diethyl pyrocarbonate
RNA	Ribonucleic acid
CA	California
USA	United States of America
BLAST	Basic Local Alignment Search Tool
DE	Differential expression
FDR	False discovery rate
DEGs	Differentially expressed genes
GO	Gene ontology
TMM	Trimmed Mean of M-values
RT-qPCR	Real-time quantitative PCR
cDNA	Complementary Deoxyribonucleic acid
MIQE	Minimum Information for Quantification Experiments in Real Time
DNA	Deoxyribonucleic acid
ANOVA	Analysis of Variance
HSD	Honest Significant Difference
NCBI	National Center for Biotechnology Information
SRA	Sequence Read Archive
TSA	Transcriptome Shotgun Assembly
BP	Biological process
CRF	Corticotropin-releasing factor
BMP	Bone morphogenetic protein

## Appendix A

**Table A1:** Toxin and venom genes identified from BLAST using Uniprot’s ToxProt database in transcriptomic sequences of *Paroctopus digueti*.

Contig ID	UniProt ID	Protein Name	GenBankIdentity %	E-Value
ASG-TRINITY_DN5661_c0_g1_i16	Q92035	Acetylcholinesterase (BfAChE) (EC 3.1.1.7)	92.87	0
ASG-TRINITY_DN3310_c3_g1_i8	W4VS99	Neprilysin-1 (EC 3.4.24.11)	90.76	0
ASG-TRINITY_DN2975_c0_g1_i1	Q75WF2	Plancitoxin-1 (EC 3.1.22.1)	85.02	0
ASG-TRINITY_DN9286_c1_g1_i11	B2D0J4	Venom dipeptidyl peptidase 4 (EC 3.4.14.5)	99.00	0
PSG-TRINITY_DN8583_c3_g1_i1	Q92035	Acetylcholinesterase (BfAChE) (EC 3.1.1.7)	92.31	0
PSG-TRINITY_DN15665_c0_g1_i1	Q8MMH3	Damage-control phosphatase ARMT1 (EC 3.1.3.-) (Venom protein 2)	78.16	0
PSG-TRINITY_DN12902_c0_g1_i1	A7ISW2	Glutaminyl-peptide cyclotransferase (EC 2.3.2.5)	100.0	0
PSG-TRINITY_DN303_c2_g1_i1	C0HKM3	Hyaluronidase conohyal-P1 (EC 3.2.1.35) (Hyaluronoglucosaminidase)	88.22	1 × 10^−104^
PSG-TRINITY_DN21310_c0_g1_i2	P0DPU3	Scoloptoxin SSD14 (SLPTX-SSD14) (Toxin-SSD14)	85.14	0
PSG-TRINITY_DN367_c0_g1_i6	Q8I6S2	Tachykinin-2 (OctTK-II) (Tachykinin II)	44.44	2 × 10^−13^
PSG-TRINITY_DN7162_c10_g1_i1	B2D0J4	Venom dipeptidyl peptidase 4 (EC 3.4.14.5)	89.68	0
PSG-TRINITY_DN135_c1_g1_i7	W8E7D1	Venom phosphodiesterase (EC 3.6.1.-)	85.41	0
DG-TRINITY_DN94241_c0_g1_i1	A3QVP0	Hyaluronidase-1	86.49	0
DG-TRINITY_DN213_c14_g1_i1	W4VS99	Neprilysin-1 (EC 3.4.24.11)	78.69	5 × 10^−168^
DG-TRINITY_DN128_c0_g1_i10	Q75WF2	Plancitoxin-1 (EC 3.1.22.1)	85.02	0
DG-TRINITY_DN37926_c0_g1_i1	A0A2I4HXH5	Snake venom 5′-nucleotidase (EC 3.1.3.5) (Ecto-5′-nucleotidase)	84.00	0
DG-TRINITY_DN1667_c0_g1_i4	W8E7D1	Venom phosphodiesterase (EC 3.6.1.-)	86.78	0
DG-TRINITY_DN1667_c0_g1_i7	J3SBP3	Venom phosphodiesterase 2 (EC 3.6.1.-)	86.78	0

**Table A2:** Antimicrobial peptide genes identified from BLAST using [22] database in transcriptomic sequences of *Paroctopus digueti*.

Contig ID	Align. Length	Name in Almeida et al., 2020 [22]	Name in GenBank	Identify %	E-Value
ASG-TRINITY_DN20858_c0_g1_i2	83	Overall_14980|DAMPD_268|11|DAMPD|DAMPD_13|ACBP_PIG	Acyl-CoA-binding protein	86.72	3 × 10^−116^
ASG-TRINITY_DN6172_c0_g1_i3	372	Overall_30231|UniProtKb_480|Q9WTV1_Q5BJR6|CH3L1_RAT	Chitinase-3-like protein 1	81.82	2 × 10^−67^
ASG-TRINITY_DN480_c1_g2_i1	97	Overall_11222|CAMP_Validated_1683|CAMPSQ4123|Histone	Histone H2A	89.71	0
ASG-TRINITY_DN18196_c0_g1_i3	102	Overall_15249|DAMPD_537|11|DAMPD|DAMPD_372|H2A_LITVA	Histone H2A	94.79	7 × 10^−166^
ASG-TRINITY_DN3219_c0_g1_i1	97	Overall_15444|DAMPD_732|11|DAMPD|DAMPD_548|H2B_LITVA	Histone H2B	92.08	0
ASG-TRINITY_DN8763_c0_g1_i1	82	Overall_15447|DAMPD_735|11|DAMPD|DAMPD_550|H4_LITVA	Histone H4	96.47	2 × 10^−141^
ASG-TRINITY_DN203799_c0_g1_i1	88	Overall_15300|DAMPD_588|11|DAMPD|DAMPD_418|LYS2_CRAVI	Lysozyme 2	98.10	1 × 10^−124^
ASG-TRINITY_DN24348_c5_g2_i1	113	Overall_11234|CAMP_Validated_1695|CAMPSQ4136|Lysozyme	Lysozyme-3-like	86.28	6 × 10^−132^
ASG-TRINITY_DN13922_c0_g1_i1	76	Overall_21373|DBAASP_5429|5781|cgUbiquitin	Ubiquitin-40S ribosomal protein S27a	99.59	1×10^-121^
ASG-TRINITY_DN246_c0_g2_i1	73	Overall_3029|APD_1148|AP02030|cgUbiquitin	Ubiquitin-40S ribosomal protein S27a	85.41	7 × 10^−131^
PSG-TRINITY_DN6816_c0_g1_i1	384	Overall_30231|UniProtKb_480|Q9WTV1_Q5BJR6|CH3L1_RAT	Chitinase-3-like protein 1	86.01	0
PSG-TRINITY_DN12886_c0_g1_i17	120	Overall_15640|DAMPD_928|11|DAMPD|DAMPD_724|H2A_ONCMY	Core histone macro-H2A.1	91.93	0
PSG-TRINITY_DN1606_c0_g1_i1	97	Overall_15444|DAMPD_732|11|DAMPD|DAMPD_548|H2B_LITVA	Histone H2B	89.22	0
PSG-TRINITY_DN60123_c0_g1_i2	108	Overall_11234|CAMP_Validated_1695|CAMPSQ4136|Lysozyme	Lysozyme 3-like	87.98	4 × 10^−124^
PSG-TRINITY_DN211390_c0_g1_i1	159	Overall_9012|CAMP_Structure_155|CAMPST238|Crystal	Peptidoglycan-recognition protein SC2-like	66.46	2 × 10^−79^
PSG-TRINITY_DN3094_c0_g1_i1	614	Overall_32035|UniProtKb_2284|P80025| PERL_BOVIN	Peroxidase-like protein	90.99	0
PSG-TRINITY_DN10374_c0_g1_i2	76	Overall_21373|DBAASP_5429|5781| cgUbiquitin	Polyubiquitin-C	99.10	0
PSG-TRINITY_DN7229_c0_g1_i1	66	Overall_32216|UniProtKb_2465|A4QNF3|ROMO1_XENTR	Reactive oxygen species modulator 1	87.88	3 × 10^−30^
DG-TRINITY_DN860_c0_g1_i12	83	Overall_14980|DAMPD_268|11|DAMPD|DAMPD_13|ACBP_PIG	Acyl-CoA-binding protein	88.49	6 × 10^−86^
DG-TRINITY_DN4732_c0_g1_i13	367	Overall_30229|UniProtKb_478|Q29411_Q5UC99|CH3L1_PIG	Chitotriosidase-1	84.19	6 × 10^−176^
DG-TRINITY_DN26923_c0_g1_i2	120	Overall_566|AMPer_566|H2A_ONCMY|Histone	Core histone macro-H2A.1	89.82	0
DG-TRINITY_DN118_c1_g1_i2	59	Overall_3101|APD_1220|AP02096| Ubiquicidin	FAU ubiquitin-like and ribosomal protein S30	91.98	2 × 10^−175^
DG-TRINITY_DN2681_c0_g1_i4	102	Overall_15249|DAMPD_537|11|DAMPD|DAMPD_372|H2A_LITVA	Histone H2A	94.92	5 × 10^−162^
DG-TRINITY_DN924_c0_g1_i4	91	Overall_15444|DAMPD_732|11|DAMPD|DAMPD_548|H2B_LITVA	Histone H2B	92.37	4 × 10^−149^
DG-TRINITY_DN118938_c0_g1_i1	82	Overall_15447|DAMPD_735|11|DAMPD|DAMPD_550|H4_LITVA	Histone H4	90.03	8 × 10^−108^
DG-TRINITY_DN1155_c0_g1_i3	113	Overall_11234|CAMP_Validated_1695|CAMPSQ4136|Lysozyme	Lysozyme	83.53	2 × 10^−168^
DG-TRINITY_DN1155_c0_g1_i2	113	Overall_31680|UniProtKb_1929|P86383|LYS_MERLU	Lysozyme	84.24	3 × 10^−126^
DG-TRINITY_DN871_c0_g2_i4	109	Overall_15299|DAMPD_587|11|DAMPD|DAMPD_417|LYS1_CRAVI	Lysozyme 1	85.17	2 × 10^−175^
DG-TRINITY_DN4246_c0_g2_i6	161	Overall_32049|UniProtKb_2298|B5T255|PGRP1_BOSIN	Peptidoglycan recognition protein 1	87.65	0
DG-TRINITY_DN3181_c0_g1_i2	463	Overall_32035|UniProtKb_2284|P80025|PERL_BOVIN	Peroxidase-like protein	88.13	0
DG-TRINITY_DN63140_c0_g1_i2	76	Overall_21373|DBAASP_5429|5781|cgUbiquitin	Polyubiquitin-C	89.66	6 × 10^−135^
DG-TRINITY_DN6534_c0_g1_i1	66	Overall_32216|UniProtKb_2465|A4QNF3|ROMO1_XENTR	Reactive oxygen species modulator 1	87.88	1 × 10^−30^
DG-TRINITY_DN2339_c1_g1_i2	165	Overall_9232|CAMP_Structure_375|CAMPST436|human	RNA exonuclease 4	71.43	1 × 10^−77^
DG-TRINITY_DN12780_c2_g3_i5	73	Overall_3029|APD_1148|AP02030|cgUbiquitin	Ubiquitin-40S ribosomal protein S27a	93.54	0
