# Supplementary material for: Tissue-Specific Gene Expression of Digestive Tract Glands in Paroctopus digueti: Insights for Cephalopod Biology and Aquaculture
Source: Animals (Basel). 2025 Nov 6;15(21):3224. doi: 10.3390/ani15213224 (PMC12607849; doi:10.3390/ani15213224)
Supplement: Supplementary file 1 [file animals-15-03224-s001.zip › animals-3894247-supplementary.pdf]

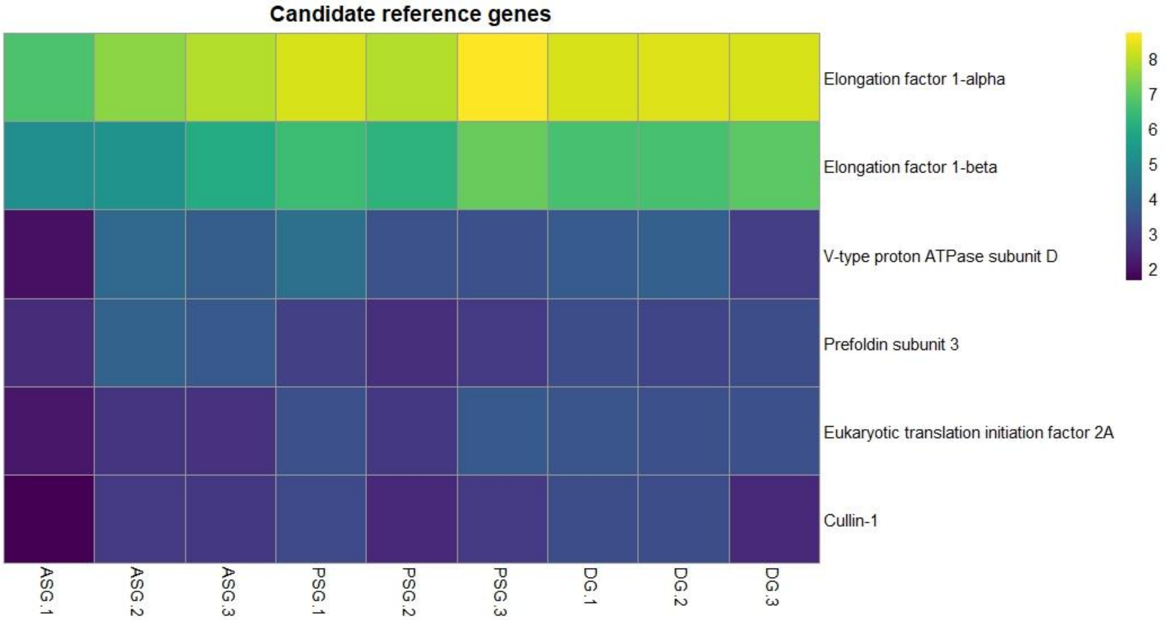

Figure S1. Candidate Reference Genes. The heatmap shows the expression of potential reference genes reported in previous works. Values represent the natural logarithm of expression levels normalized by the TMM method. The color scale indicates maximum expression in yellow, moderate in green, and low in dark blue.
